# Gender differences in correlation of biochemical parameters with the severity of covid pneumonia and the need for oxygen/mechanical support

**DOI:** 10.5937/jomb0-49377

**Published:** 2025-03-21

**Authors:** Jelena Jankovic, Mihailo Stjepanovic, Nikola Maric, Slobodan Belic

**Affiliations:** 1 University Clinical Center of Serbia, Clinic for Pulmonology, Belgrade; 2 University of Belgrade, Medical Faculty, Belgrade

**Keywords:** COVID-19, gender, biochemical results, mechanical ventilation, oxygen therapy, KOVID-19, pol, biohemijske analize, mehanička ventilacija, kiseonična terapija

## Abstract

**Background:**

The COVID-19 pandemic caused global medical, economic and social problems. High infection rates, heterogeneous presentation, lack of previous data, and lack of standardized treatment led to a need for further analysis to prepare for potential new pandemics. We analyzed any possible correlation between gender, laboratory findings, disease severity and the need for oxygen or mechanical ventilation support.

**Methods:**

99 patients with confirmed SARS-CoV-2 virus infection enrolled. Baseline characteristics that included age, sex, smoking history, BMI, oxygen therapy or mechanical ventilation support needs were recorded. Type and severity of radiological findings determined by chest CT scan.

**Results:**

The majority of our patients were over 61 years old (58.6%), male (57.6%), and had severe radiological findings (bilateral pneumonia 29.3%, ARDS 35.4%), with only 20.2% had not required any oxygen supplementation. Regarding gender and laboratory findings, men have shown statistically significant higher values of CRP, lymphocytes, LDH and ferritin (96.4 vs 87.1, p=0.014; 1.17 vs 0.84, p=0.048; 674.8 vs 609.1, p=0.031; 1263 vs 578.4, p=0.001, respectfully). Severe radiological findings showed a positive correlation with the need for HFNC and/or (N)IMV (p=0.021 and p=0.032. respectfully), as well as with higher values of WBC, LDH and ferritin (p=0.042, p=0.035 and p=0.017, respectively).

**Conclusions:**

There is a difference between the presentation of the disease and analyzed laboratory markers between sexes. The difference is most likely multifactorial and should require further research in order to discover other risk and prognostic factors.

## Abstract (sr)

**Uvod:**

Pandemija KOVID-19 je izazvala globalni medicinski, ekonomski i socijalni problem. Visoka stopa zaraze, heterogena klinička slika, nedostatak prethodnih podataka i nepostojanje standardizovanog protokola lečenja su doveli do potrebe za daljom analizom, kako bi se ovi problem prevazišli u slučaju potencijalne nove pandemije. Analizirali smo moguću korelaciju između pola, laboratorijskih nalaza i tezine bolesti sa potrebom za kiseonikom ili pušački status.

**Metode:**

Uključeno je 99 pacijenata sa potvrđenom infekcijom virusom SARS-CoV-2. Zabeležene su osnovne karakteristike koje su uključivale starost, pol, pušački status, BMI, terapiju kiseonikom ili potrebu za mehaničkom ventilacionom potporom. Vrsta i težina radiografskog nalaza utvrđena je skenerom grudnog koša.

**Rezultati:**

Većina naših pacijenata je bila starija od 61 godine (58,6%), muškog pola (57,6%) i imali su teške radiografske manifestacije KOVID-19 infekcije (bilateralna pneumonija 29,3%, ARDS 35,4%), a samo 20,2% nije zahtevalo dodatnu primenu kiseonične terapije. U pogledu pola i laboratorijskih nalaza, muškarci su pokazali statistički značajno veće vrednosti CRP, limfocita, LDH i feritina (96,4 vs 87,1, p=0,014; 1,17 vs 0,84, p=0,048; 674,8 vs 609,1, p=0,031; 1263 vs 578,4, p=0,001). Teži radiološki nalaz pokazao je pozitivnu korelaciju sa potrebom za HFNC i/ili (N)IMV (p=0,021 i p=0,032), kao i sa višim vrednostima leukocita, LDH i feritina (p=0,042, p=0,035 i p=0,017).

**Zaključak:**

Prisutne su razlike između kliničke manifestacije bolesti i analiziranih laboratorijskih markera između polova. Razlika je najverovatnije višefaktorna i zahteva dalja istraživanja kako bi se otkrili drugi prognostički i faktori rizika.

## Introduction

COVID-19 is a systemic infectious disease caused by the SARS-CoV-2 coronavirus. The most common infection transmission route is the droplet route, and depending on the virus variant, the incubation period can be from several days to up to two weeks [1] [2]. The majority of patients have asymptomatic or mild disease with symptoms of the upper respiratory system, while approximately 20% have severe disease [3]. For the past few years, COVID-19 has been a worldwide health problem with an impact on morbidity and mortality, but also social reasons for concern. Thanks to the development of new antiviral and biological therapies and vaccines, the emergency pandemic was soon abolished, and the number of patients and deaths decreased. However, the epidemic continues, and we still encounter severe cases that are treated in the intensive care unit (ICU) and a certain percentage end with a fatal outcome [4]. The virus invades and destroys alveolar cells and causes a systemic inflammatory response with a cytokine storm, followed by fibrosis [5] [6]. Over 15% of hospitalized patients develop acute respiratory distress syndrome (ARDS). These patients require mechanical ventilation support or high-flow oxygen therapy via a highflow nasal cannula (HFNC) up to 60 litres per minute [7].

Laboratory diagnostics of COVID-19 are important in establishing a diagnosis and determining the disease stage, prognosis, therapeutic monitoring, and epidemiological surveillance. Identifying prognosis predictors is important when choosing different treatment modalities. Many prognostic factors have been described in the literature data from previous studies, such as the levels of interleukin 6 (IL- 6), C-reactive protein (CRP), ferritin, lymphocytes (LYM) and D-dimer [8]. However, their role in viral infections and systemic inflammation remains unclear. The role of LYM in viral infections is well-known [9]. Also, there is a question of whether there are gender differences in the incidence and characteristics of COVID-19 pneumonia.

We hypothesized that other biomarkers, except LYM, can predict the severe clinical and radiological presentation and need for oxygen or (none)invasive mechanical ventilation support (N)(IMV). This study aimed to evaluate and identify the most effective, accessible and predictive biomarkers that could be used in clinical practice in COVID pneumonia, the need for oxygen\mechanical ventilation support, and better risk stratification of the patients and to see if gender differences exist.

## Materials and methods

### Patient characteristics and data collection

Retrospective study was conducted at the Clinic for Pulmonology, University Clinical Center of Serbia in 2021. The study included 99 patients with confirmed SARS-CoV- 2 virus infection. The study follows the Declaration of Helsinki and the Institutional Review Board of the University of Belgrade (protocol no. 6711\2021).

The inclusion criteria were having a confirmed COVID-19 infection through RT-PCR or antigen testing, being older than 18 years, and being admitted to the hospital and treated in the SICU or ICU. Upon admission, patients signed an informed consent. Data retrieved from electronic medical records included the sociodemographic information, clinical characteristics (COVID-19 symptoms upon hospital admission), need for oxygen therapy, HFNC, NIMV or MV), chest X-ray imaging methods, and laboratory analyses (complete blood count, lactate dehydrogenase- LDH, C-reactive protein, ferritin).

### Statistical analysis

Statistical analysis was performed using the Statistical Package for Social Sciences version 21. The significance level was set at 0.05. The study sample was described using means and standard deviations for continuous variables, and the results were compared using the t-test for independent samples. Categorical variables are expressed as frequency and percentage and were compared using Pearson’s 2 test or Fisher’s exact test, as appropriate. Spearman’s correlation coefficient was calculated to assess the correlation between the variables of interest – data presented with tables and graphics.

## Results

**Figure 1:**
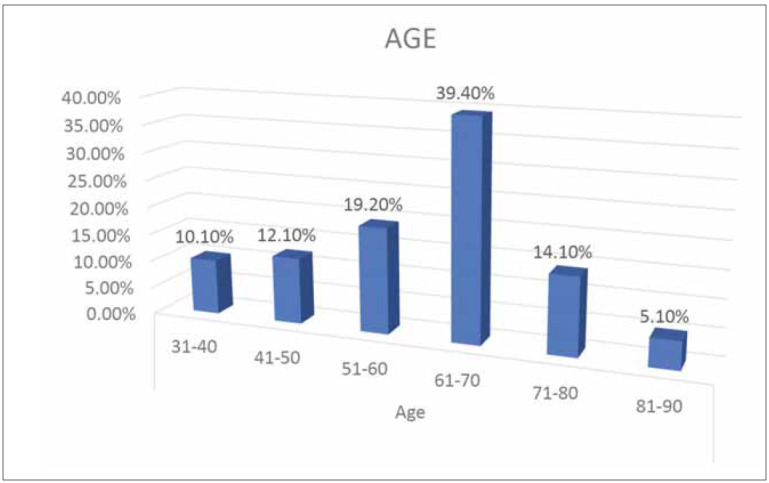
The distribution of patients according to age.

We included 99 hospitalized adult patients with positive RT-PCR results for SARS-CoV-2 in our study. The median age of the patients was 60.0±12.4 years, and 42 (42.4%) were female. The distribution of patients according to age is illustrated in Figure 1. The highest number of patients, about 40%, was in the age category 61–70 years, followed by the 51–60 years category.

The radiological chest X-ray findings and the need for using oxygen or mechanical support are shown in Table 1. Only two patients had no changes on the chest radiography. Distribution of pathological structural changes was: 24% of g00 patients had accentuated interstitial pattern, 9 (9.1%) had unilateral, and 29 patients (29.3%) had bilateral pneumonia, while 35 (35.4%) patients had diffuse changes as ARDS. Of the 99 patients, 64 (64.6%) had severe disease according to structural changes, and 35 (35.4%) had nonsevere disease. Radiographic chest findings compared by gender showed higher prevalence and a statistically significant difference in unilateral (p 0.003) and accentuated interstitial pattern (p 0.017) in female patients (Table 2).

**Table 1:** Distribution of patients according to chest radiography findings and use of oxygen therapy or mechanical support.

		n (%)
Radiological finding	No changes	2 (2)
	Accentuated interstitial	24 (24.2)
	Unilateral pneumonia	9 (9.1)
	Bilateral pneumonia	29 (29.3)
	ARDS	35 (35.4)
O2/MV	Without	20 (20.2)
	Mask	53 (53.5)
	HFNC	16 (16.2)
	NIMV	9 (9.1)
	MV	1 (1)

**Table 2:** Differences in results between the genders.

N (%)	Female 41 (41.4)	Men 58 (58.6)	p
Age	62.5±11.2	60.2±12.1	0.334
CRP	87.1	96.4	0.014
WBC x10^9^/L	7.11	7.13	0.781
LYM x10^9^/L	0.84	1.17	0.048
LDH	609.1	674.8	0.031
Ferritin	578.4	1263.2	0.001
BMI	21.4	24.8	0.006
Radiological finding <br>*No changes <br>*Accentuated interstitial pattern <br>*Unilateral pneumonia <br>*Bilateral pneumonia <br>*ARDS	<br>0 <br>9 (23.8) <br>5 (11.9) <br>12 (28.6) <br>15 (35.7)	<br>3 (5.2) <br>15 (25.8) <br>3 (5.2) <br>17 (29.3) <br>20 (34.5)	0.011
Oxygen or MV <br>*Without <br>*Mask <br>*HFNC <br>*NIMV <br>*MV	<br>9 (21.4) <br>20 (47.6) <br>9 (21.4) <br>3 (9.6) <br>0	<br>11 (18.9) <br>33 (56.9) <br>7 (21.1) <br>6 (10.4) <br>1 (1.7)	

Three quarters of patients were on oxygen therapy; half had oxygen therapy by mask, 16 were on HFNC, nine were on NIVM, and only one was on mechanical ventilation support (Table 1). Compared by gender, we showed higher prevalence and a statistically significant difference in oxygen by mask (p 0.032), NIMV (p 0.001) and MV in male patients (Table 2).

Body mass index (BMI) for both genders was in the referent range for normal BMI, but for men, it was on the upper limit and near overweight.

Two-thirds of the patients had white blood cell counts (WBC) within the reference range, 12% had decreased values, and 19% had elevated values. Two-thirds of the patients had lymphocyte counts decreased. CRP values were elevated in 96% of the study population. More than 80% of the patients had elevated LDH values, and ferritin was elevated in two-thirds of the patients. Statistically significant differences in biochemical values, with higher values in the male population, are presented in Table 2.

Table 3 presents correlation radiological findings with BMI, oxygen, MV support, and biochemical values. A negative correlation existed between radiological findings (bilateral pneumonia or diffuse lung damage) during infection with FHNC therapy using NIV or MV. A negative correlation between leukocyte values during infection and severe radiological findings was noted. Patients with higher WBC values had significantly severe structural changes. There was no statistically significant correlation between the values of lymphocytes and CRP during the infection. There was a weak, negative correlation of LHD and ferritin during infection with severe radiological findings. Patients with higher LDH and ferritin values had a significantly more severe form of pneumonia (bilateral and ARDS).

**Table 3:** Correlation between severe radiological findings and other factors of interests.

	Severe radiographic findings
	p
Oxygen	0.046
HFNC	0.021
(N)IMV	0.032
WBC	0.042
#LYM	0.923
CRP	0.031
LDH	0.035
Ferritin	0.017
BMI	0.027

There was no lethal outcome; all patients were discharged home after hospital treatment for an average of 11 days. The longest duration of hospitalization was 31 days.

## Discussion

In this study, we want to investigate the underlying reasons for the gender-based differences in COVID-19 infection and provide our views for future research. According to data collected by the World Health Organization (WHO), the number of patients treated because of COVID-19 infection is similar in both sexes [10]. However, contrary to this data, a meta-analysis of Amgalan and colleagues showed that male patients are more likely to experience severe disease and poor outcomes compared with females [11]. More than half (nearly 60%) of patients in our study were men, following meta-analysis data. Men’s higher prevalence can be underlying biological differences, habits or behavioural differences [11]. All countries providing COVID-19 admission rates by sex reported higher hospitalization rates in males than in females [12]. Gender-based differences include women’s higher frequency of physician visits compared to men’s and women’s greater adherence to everyday therapy, checkups and personal hand hygiene that was recommended during the pandemic [13].

In contrast, men are more likely to engage in risky behaviour with activities that may compromise their health, frequent alcohol use and smoking [13]. Also, according to previous research data, the X chromosome confers females a significant immunological advantage over males; they have stronger antibody and cell-mediated immune responses [11] [14]. Studies have found an association between lower testosterone levels and increased inflammatory cytokines, so reduction of testosterone in older men may be responsible for severe disease. In females, reduced estrogen levels in menopause have increased infection risk. Estrogens are considered immune-stimulatory and thus may be protective in COVID-19 [15] [16]. Our study data, with an average age of patients over 50 with physiologically reduced sex hormone values and menopause in women, also support this theory.

Biomarker levels are reported by sex, but data about the effect of patient sex on the relationship between biomarkers and COVID-19 disease severity are scarce. It is proved that LYM is among the first responders to viral agents. Lymphopenia is a sign of a reduced immune response to viral infections such as COVID. Recent literature shows that they are also associated with COVID-19 severity [17]. The presumed reason for lymphopenia is that in the severe form of COVID-19, there is an increase in the total number of WBCs, and the percentage of LYM is reduced [18]. Meta-analysis of the mean difference in admission lymphocyte counts between patients with and without severe COVID-19 outcomes showed that lymphopenia and disease severity were not modified by sex or co-morbidities [19]. However, some previous COVID-19 studies showed that male patients are inversely associated with lymphocyte count [17]. We found the statistical borderline significance of lower LYM count in male patients correlated with severe disease and frequent use of oxygen therapy by mask, NIMV and MV support.

In a retrospective study of one centre conducted on 145 patients with COVID-19, CRP was defined as an early detector of disease severity and an adequate biomarker for therapy planning [20]. Also, there are studies where no significant difference in CRP level was observed comparing patients with mild, severe, and critical clinical pictures, following our results [21]. However, there was a statistically significant difference in values of CRP in male and female patients, which can be a consequence of severe clinical presentation of disease in men.

Ferritin was increased in two-thirds of study patients and statistically significantly higher about two times in male patients. The theory about ferritin as a product of inflammation and that hyper-ferritinemia, caused by excessive inflammation in infection, is significantly represented. It is shown that increased levels of ferritin are associated with severe clinical presentation, high mortality and ICU admission [22] [23]. That can be an explanation for differences in gender values in our study. Male patients had a higher number of patients who needed NIM or MV support and needed to be treated in the ICU.

Many studies investigate the effects of BMI and obesity on clinical outcomes of COVID-19. Obesity increases the risk of hospitalization, severe disease form, ICU admission, need for MV support and death among patients with COVID-19 infection [24]. Overweight people tend to have respiratory dysfunction, a higher oxygen cost of breathing is needed for them, shortness of breath was found to be the leading clinical symptom, and they can develop hypoxaemia with hypercapnia. Because of that, they are at a greater risk of needing MV support and admission to ICU [25] [26]. Even though the BMI for both genders in our study population was in the referent range for normal BMI, for male patients, it was on the upper limit, near being overweight (BMI 24.8). That is why particular caution is needed in these male patients, and this is the reason for the higher number of ICU admissions and the need for (N)IMV support in men. The findings in our study are consistent with previous reports, suggesting a positive relationship between higher BMI and increased risk of disease severity among patients with COVID-19 and ICU admission.

This study has a few limitations. The first is that only one centre was included without comparing results with another centre. Second, there was a small number of mechanical ventilation patients. Third is the impossibility at that moment of processing the hormonal status of sex hormones as a maybe reason for differences between genders in the clinical presentation of COVID-19 infection.

## Conclusion

Male and female patients present significant differences regarding COVID-19 infection. The higher COVID- 19 severity disease rate in males than females is likely due to genetic, hormonal, co-morbidities and immunity. We conclude that sex differences may affect the pathogenic mechanisms of COVID-19 and the severity of the disease, values of biomarkers, radiological findings, and need for MV support. Females generally have a stronger immune response; males are more likely to develop the cytokine storm associated with poor clinical presentation. Further investigation into gender differences and underlying mechanisms may help explain the worse survival of men.

## Dodatak

### Acknowledgement

All authors have contributed equally towards the conception of the manuscript.

### Authors’ Contributions

All authors contributed to the study’s conception and design. Jelena Jankovic and Slobodan Belic performed data collection and analysis. Statistical analysis Nikola Maric. Jelena Jankovic wrote the first draft of the manuscript, and all authors commented on previous versions. Critical revision of the manuscript: Andjelka Ivanovic. All authors read and approved the final manuscript.

### Funding

This research was funded by the Ministry of Education, Science and Technological Development of the Republic of Serbia (Project No. 200110).

### Conflict of interest statement

All the authors declare that they have no conflict of interest in this work.
